# Supplementary material for: Aromas: Lovely to Smell and Nice Solvents for Polyphenols? Curcumin Solubilisation Power of Fragrances and Flavours
Source: Molecules. 2024 Jan 5;29(2):294. doi: 10.3390/molecules29020294 (PMC10820666; doi:10.3390/molecules29020294)
Supplement: Supplementary file 1 [file molecules-29-00294-s001.zip › molecules-2718089-supplementary.pdf]

Supplementary Information

Aromas: Lovely to Smell and Nice Solvents for Polyphenols? Curcumin Solubilisation Power of Fragrances and Flavours †

Michael Schmidt [1*], Verena Huber [2], Didier Touraud [2] and Werner Kunz [2,*]

[1] Institute of Materials Resource Management, University of Augsburg, Am Technologiezentrum 8, D-86159 Augsburg, Germany
2 Institute of Physical and Theoretical Chemistry, University of Regensburg, D-93040 Regensburg, Germany; verena1.huber@chemie.uni-regensburg.de (V.H.); didier.touraud@chemie.uni-regensburg.de (D.T.)
* Correspondence: michael1.schmidt@uni-a.de (M.S.); werner.kunz@chemie.uni-regensburg.de (W.K.)
† This paper is dedicated to Farid Chemat, an outstanding scientist, colleague, and friend. Rest in peace, cher ami.

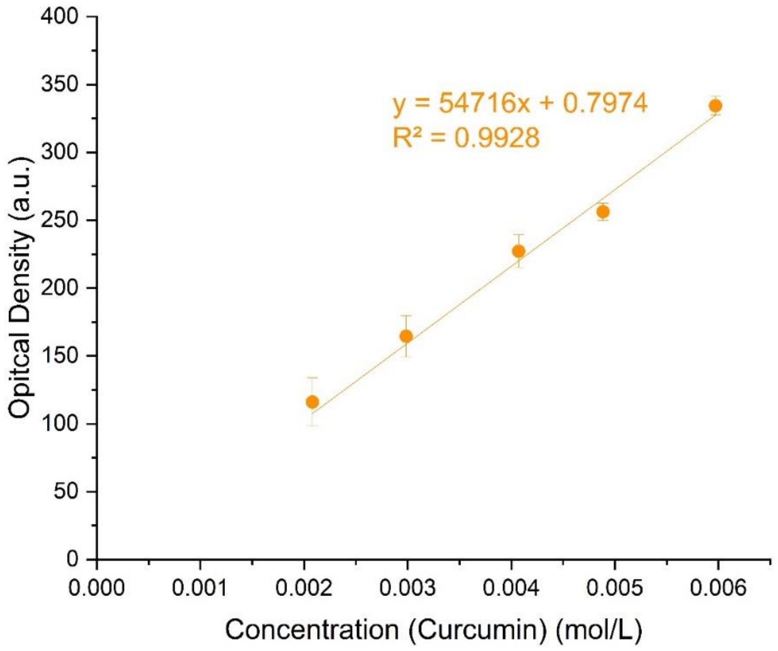

Figure S 1 Calibration curve of curcumin in ethanol at λ=425 nm.

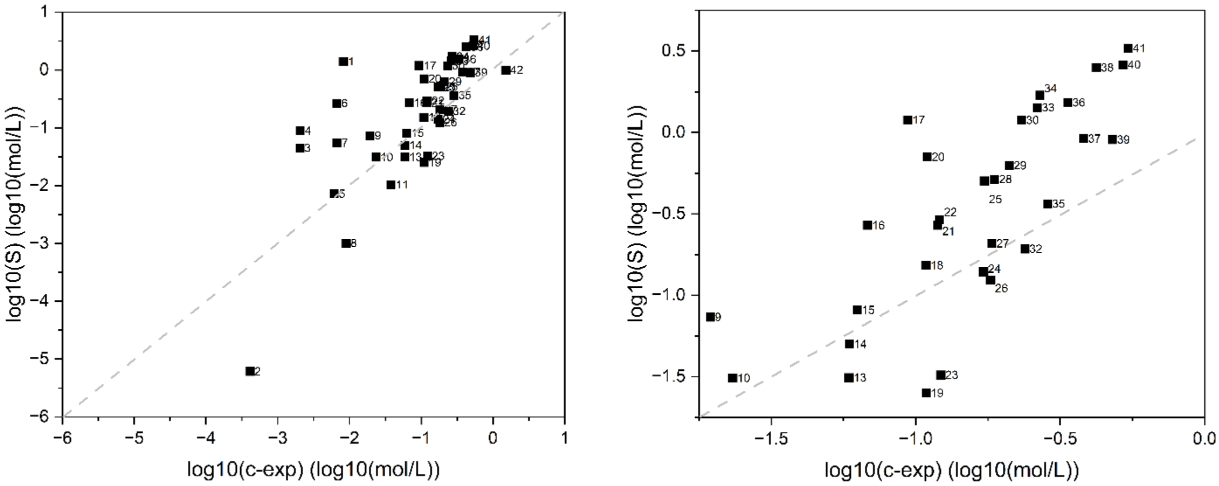

(a) **(b)**

Figure S 2 Calculated solubility of curcumin log10(S) vs the logarithmic curcumin concentration c(x) in the respective liquid solvents, (a) showing the whole graph, while (b) shows the section between -2<log10(c(x))<0 for better identification of the datapoints. The numbering of labels refers to the list in Table 1. Vanillin and veratraldehyde are excluded due to being solid compounds.

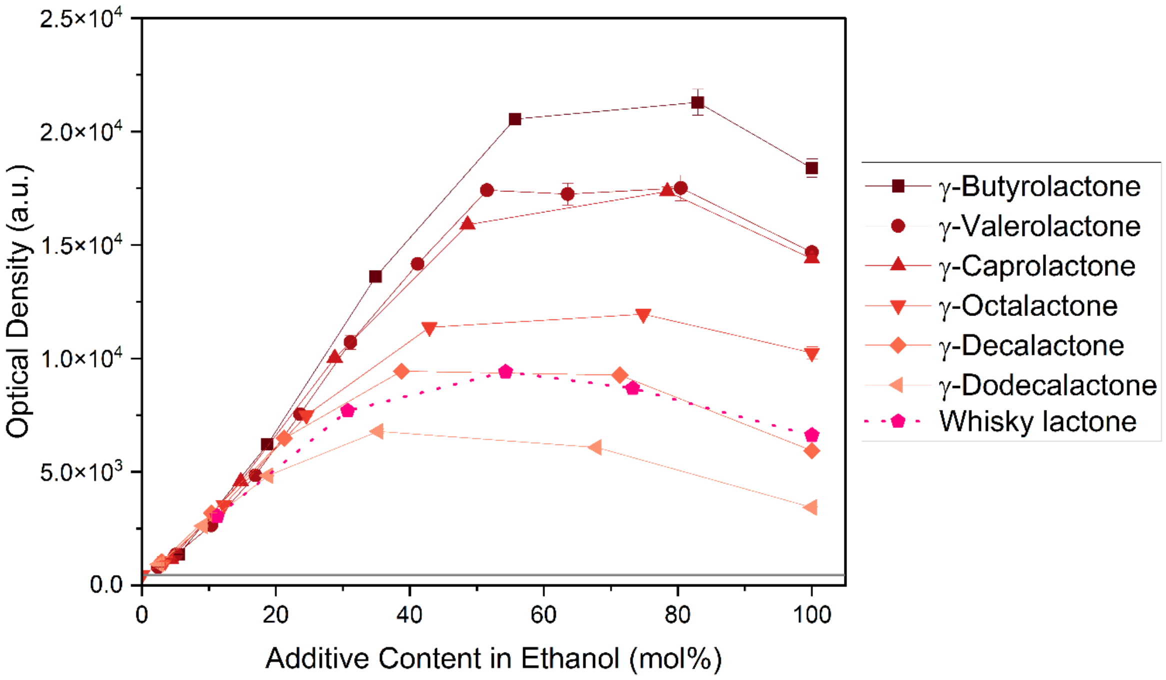

Figure S 3 Optical densities in arbitrary units (a.u.) of curcumin in binary ethanolic mixtures with γ-lactones.

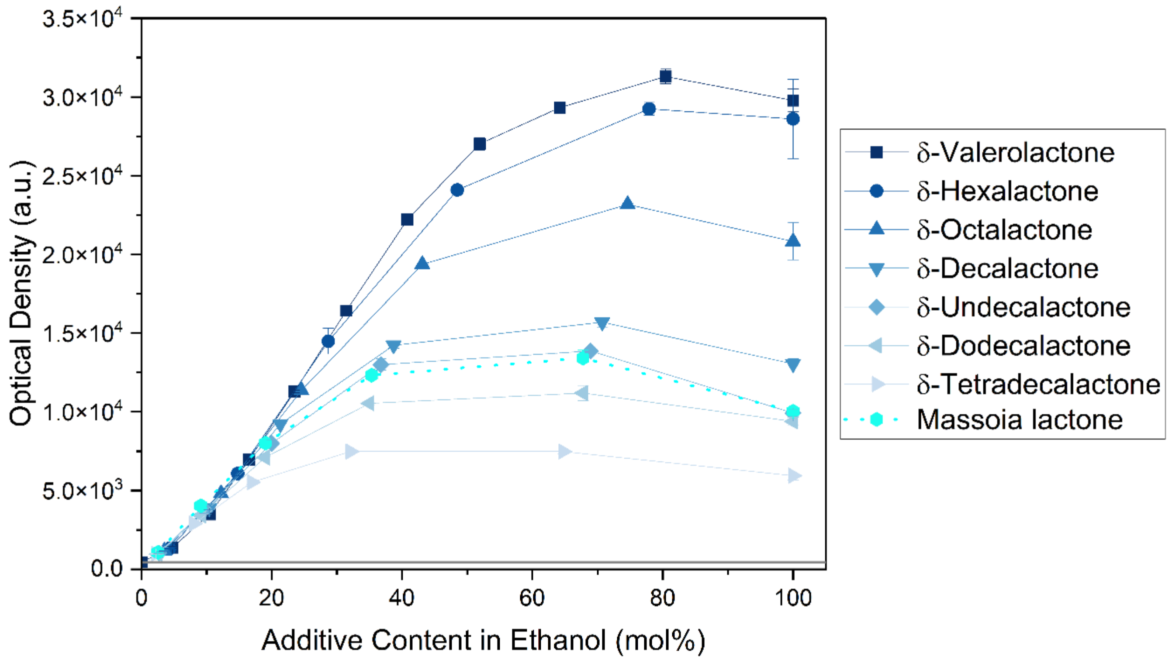

Figure S 4 Optical densities in arbitrary units (a.u.) of curcumin in binary ethanolic mixtures with δ-lactones.

(a)

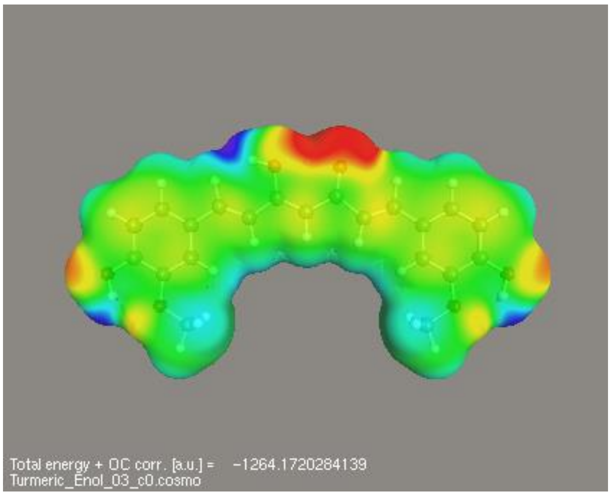

(b)

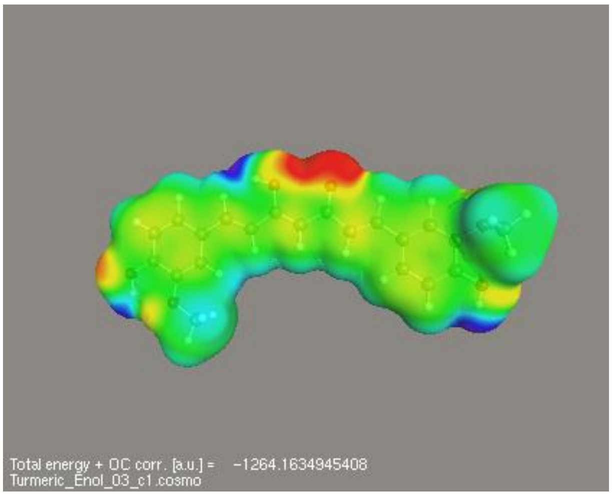

(c)

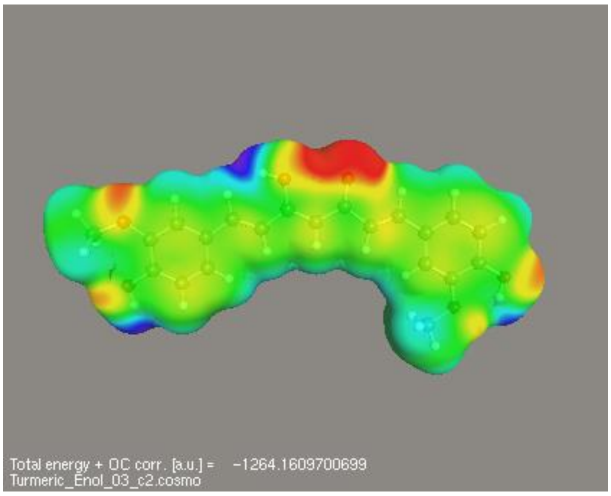

(d)

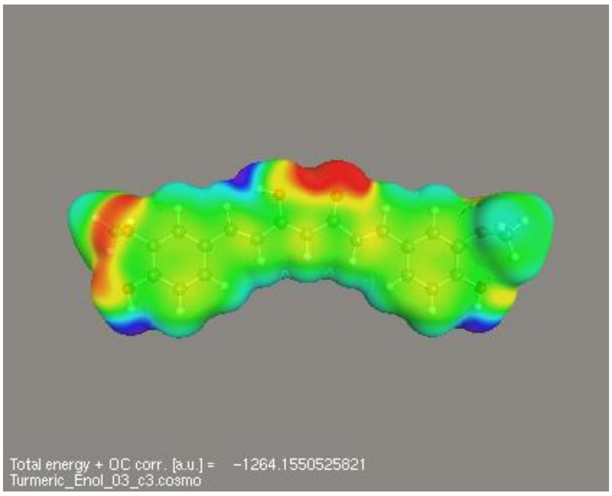

Figure S 5 COSMO surfaces of the four keto-enol conformers of curcumin, (a) conformer 1, (b) conformer 2, (c) conformer 3, and (d) conformer 4.

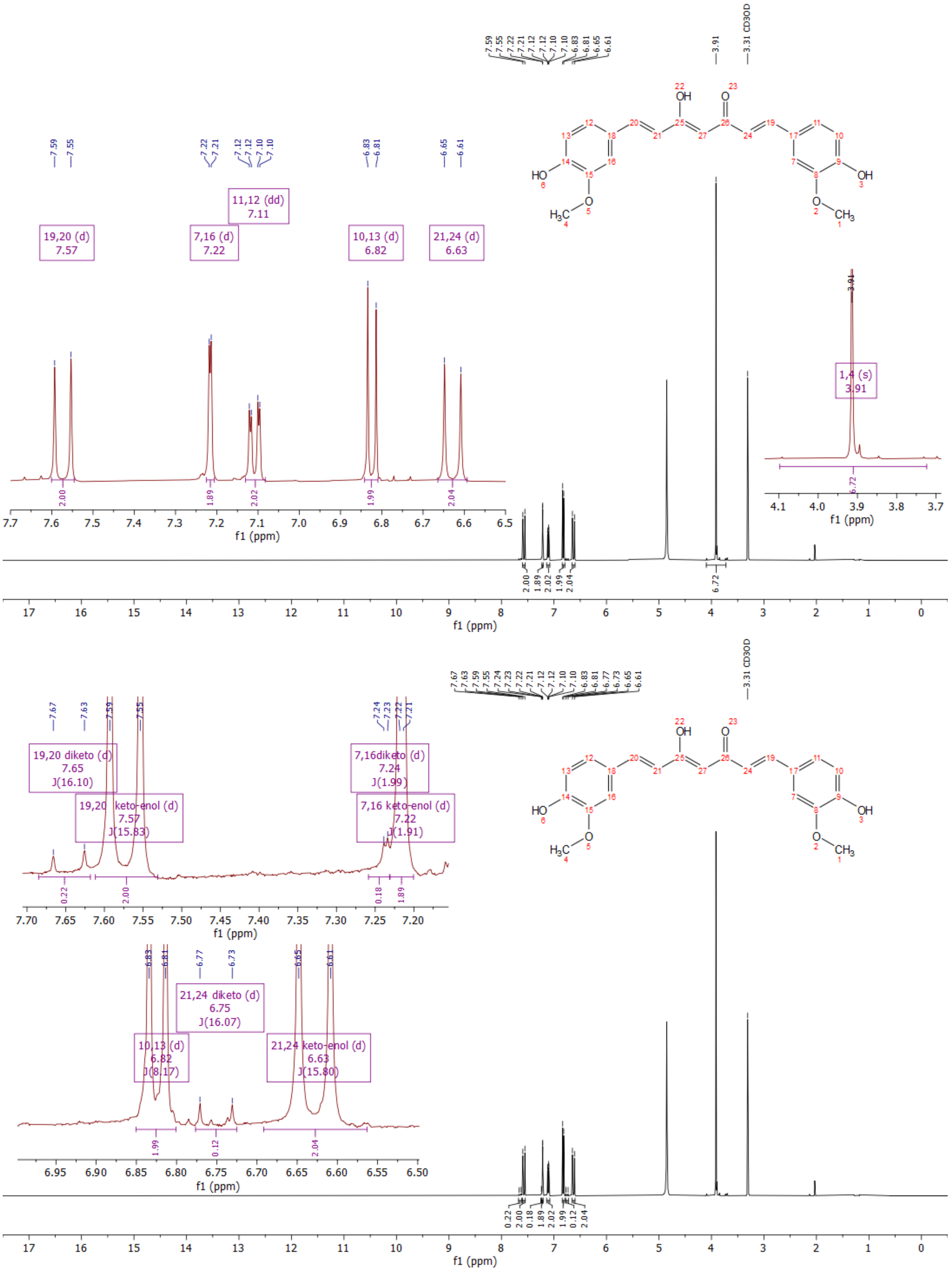

Figure S 6 ¹H-NMR spectrum of curcumin in methanol-d4 with assigned signals to the predominant conformer of curcumin (top) and of diketo/keto-enol groups of curcumin (bottom).

Signals of curcumin: δ H (400 MHz, methanol-d4) 3.91 (7 H, s), 6.63 (2 H, d, J 15.8), 6.82 (2 H, d, J 8.2), 7.11 (2 H, dd, J 8.2, 1.9), 7.22 (2 H, d, J 1.9), 7.57 (2 H, d, J 15.8).

Signals of diketo/keto-enol groups of curcumin: δ H (400 MHz, methanol-d4) 6.63 (2 H, d, J 15.8), 6.75 (0 H, d, J 16.1), 6.82 (2 H, d, J 8.2), 7.11 (2 H, dd, J 8.2, 1.9), 7.22 (2 H, d, J 1.9), 7.24 (0 H, d, J 2.0), 7.57 (2 H, d, J 15.8), 7.65 (0 H, d, J 16.1).

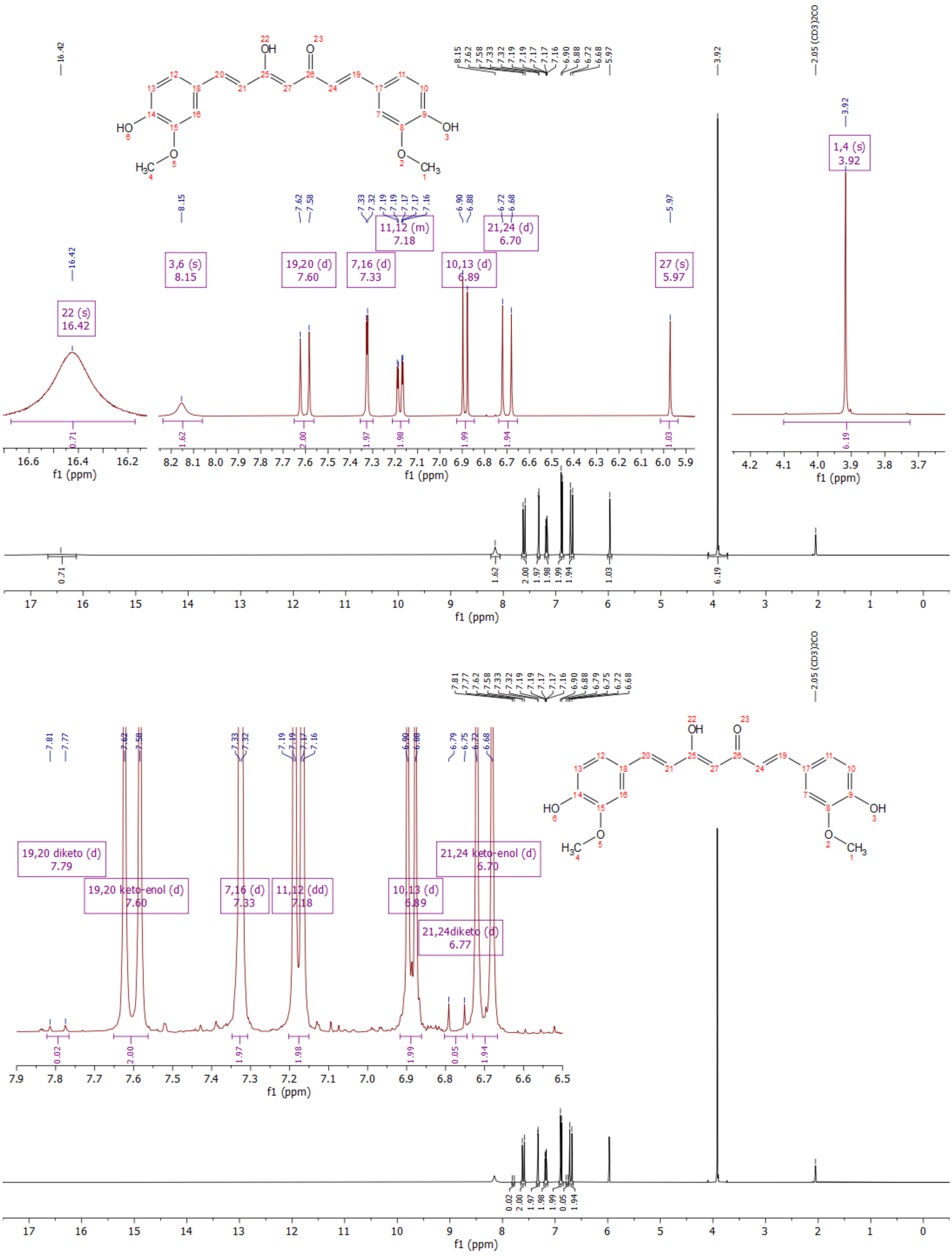

Figure S 7 ^{1}H-NMR spectrum of curcumin in acetone-d6 with assigned signals to the predominant conformer of curcumin (top) and of diketo/keto-enol groups of curcumin (bottom).

Signals of curcumin: δ H (400 MHz, acetone-d6) 3.92 (6 H, s), 5.97 (1 H, s), 6.70 (2 H, d, *J* 15.8), 6.89 (2 H, d, *J* 8.2), 7.14 – 7.21 (2 H, m), 7.33 (2 H, d, *J* 1.9), 7.60 (2 H, d, *J* 15.8), 8.15 (2 H, s), 16.42 (1 H, s).

Signals of diketo/keto-enol groups of curcumin: δ_H (400 MHz, acetone-d6) 3.92 (6 H, s), 5.97 (1 H, s), 6.70 (2 H, d, *J* 15.8), 6.77 (0 H, d, *J* 16.1), 6.89 (2 H, d, *J* 8.2), 7.18 (2 H, dd, *J* 8.2, 2.0), 7.33 (2 H, d, *J* 1.9), 7.60 (2 H, d, *J* 15.8), 7.79 (0 H, d, *J* 15.9), 8.15 (2 H, s), 16.42 (1 H, s).

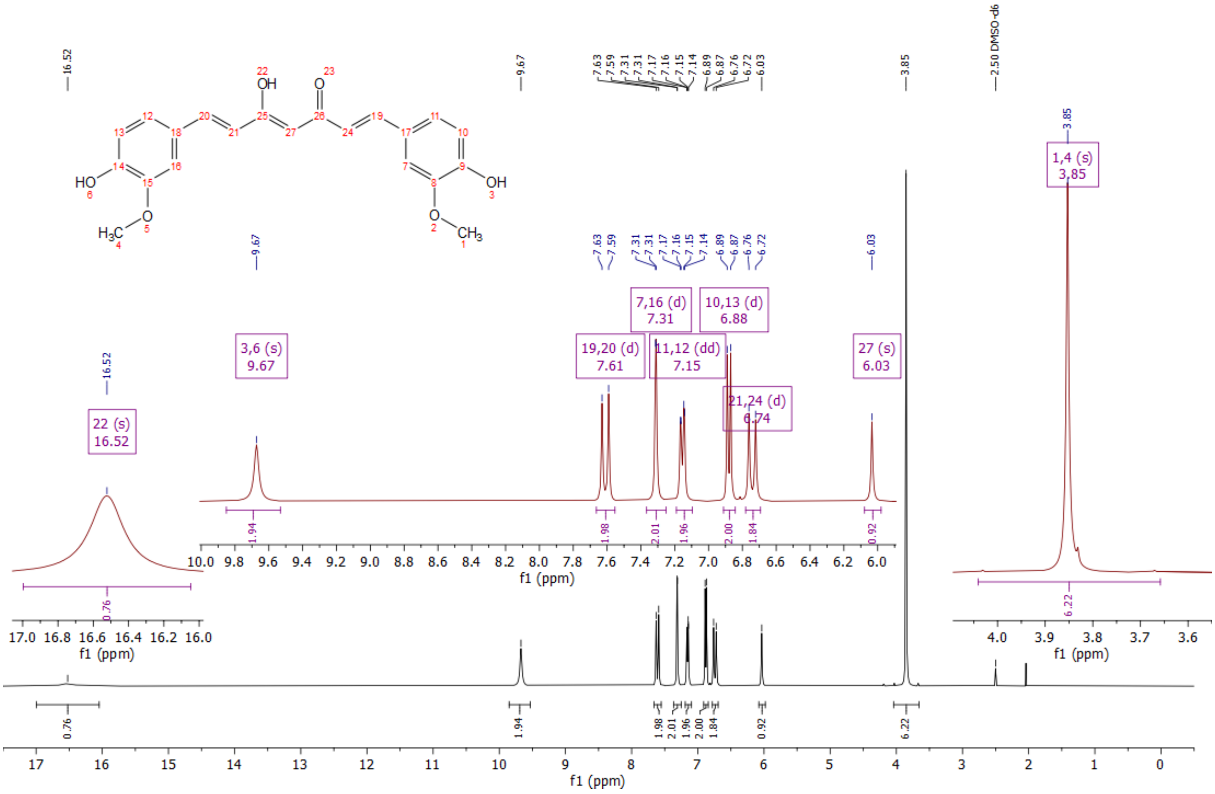

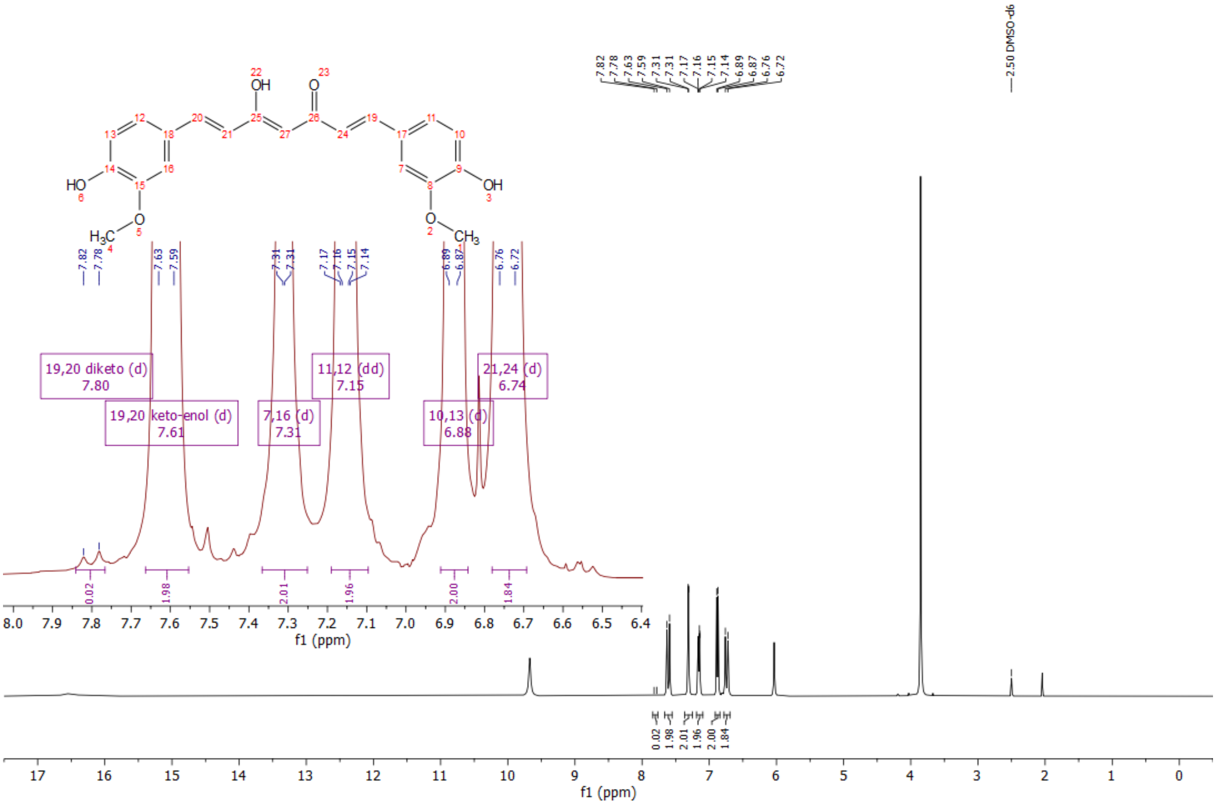

Figure S 8 ^{1}H-NMR spectrum of curcumin in DMSO-d6 with assigned signals to the predominant conformer of curcumin (top) and of diketo/keto-enol groups of curcumin (bottom).

Signals of curcumin: δ H (400 MHz, DMSO-d6) 3.85 (6 H, s), 6.03 (1 H, s), 6.74 (2 H, d, *J* 15.7), 6.88 (2 H, d, *J* 8.1), 7.15 (2 H, dd, *J* 8.4, 1.8), 7.31 (2 H, d, *J* 1.9), 7.61 (2 H, d, *J* 15.6), 9.67 (2 H, s), 16.52 (1 H, s).

Signals of diketo/keto-enol groups of curcumin: δ H (400 MHz, DMSO-d6) 6.74 (2 H, d, *J* 15.7), 6.88 (2 H, d, *J* 8.1), 7.15 (2 H, dd, *J* 8.4, 1.8), 7.31 (2 H, d, *J* 1.9), 7.61 (2 H, d, *J* 15.6), 7.80 (0 H, d, *J* 15.7).

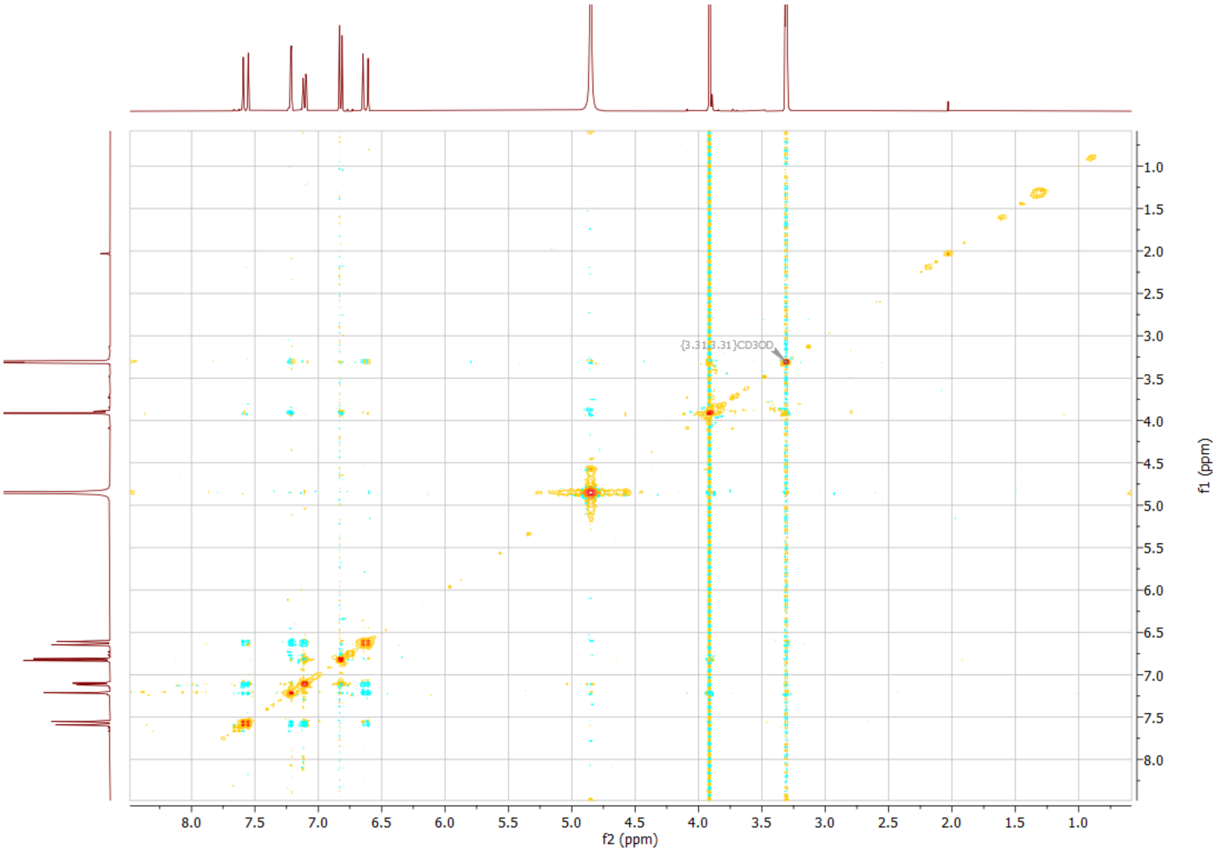

Figure S 9 ^{1}H-^{1}H NOESY spectrum of curcumin in methanol-d4.

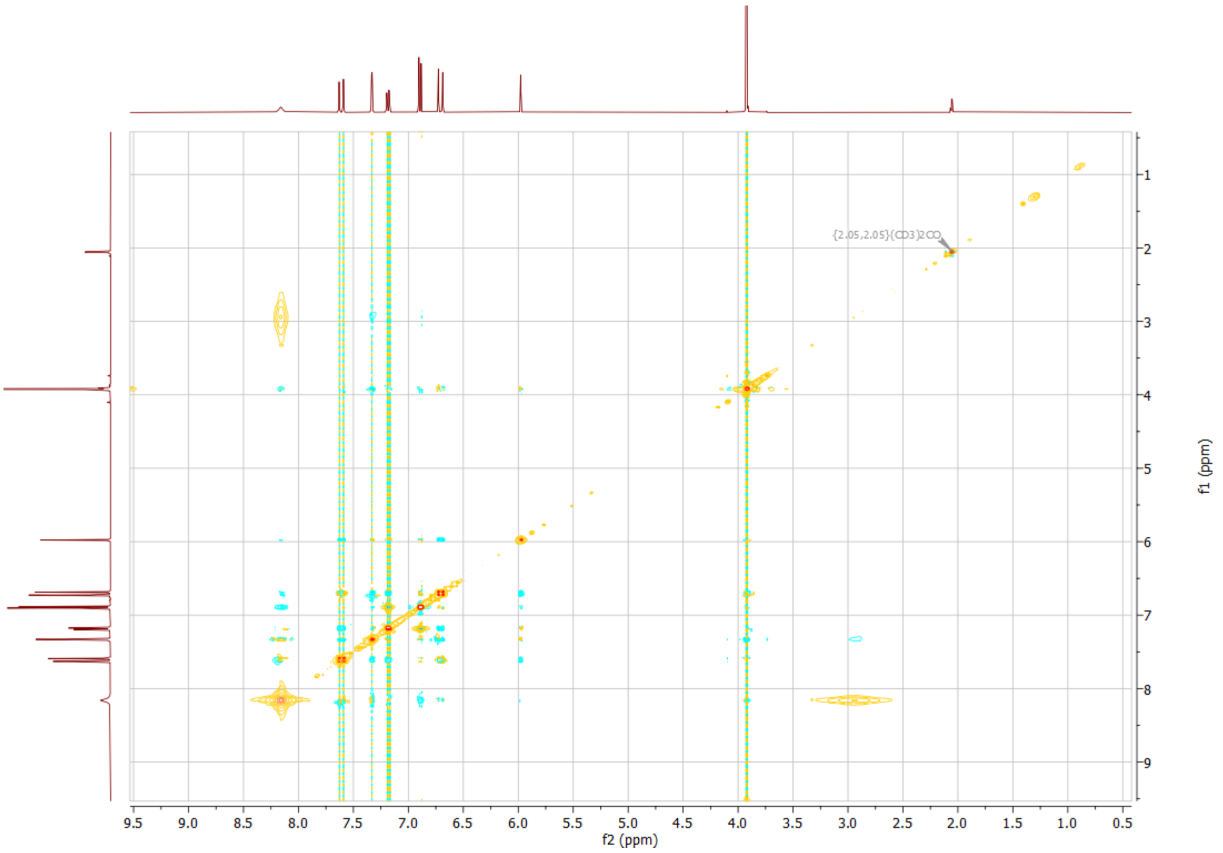

Figure S 10 ^{1}H-^{1}H NOESY spectrum of curcumin in acetone-d6.

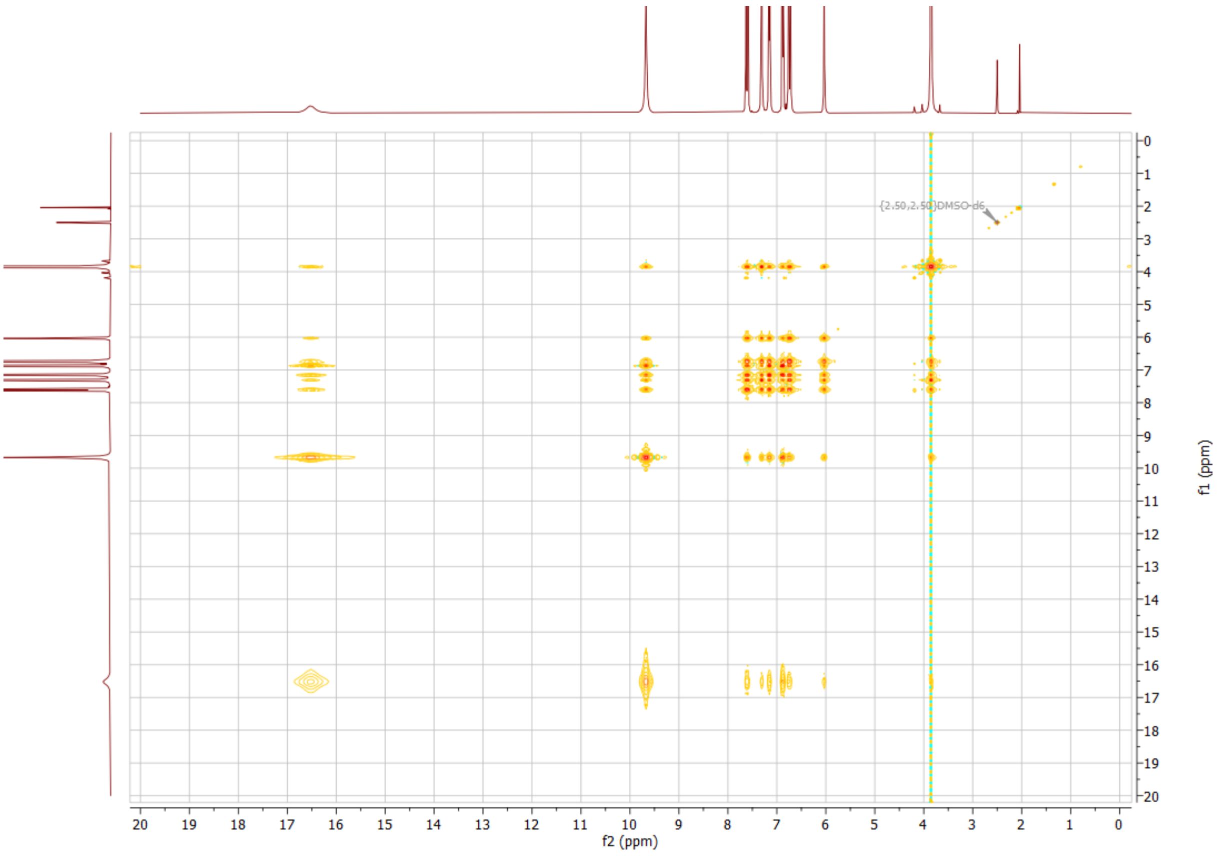

Figure S 11 ^{1}H-^{1}H NOESY spectrum of curcumin in DMSO-d6.

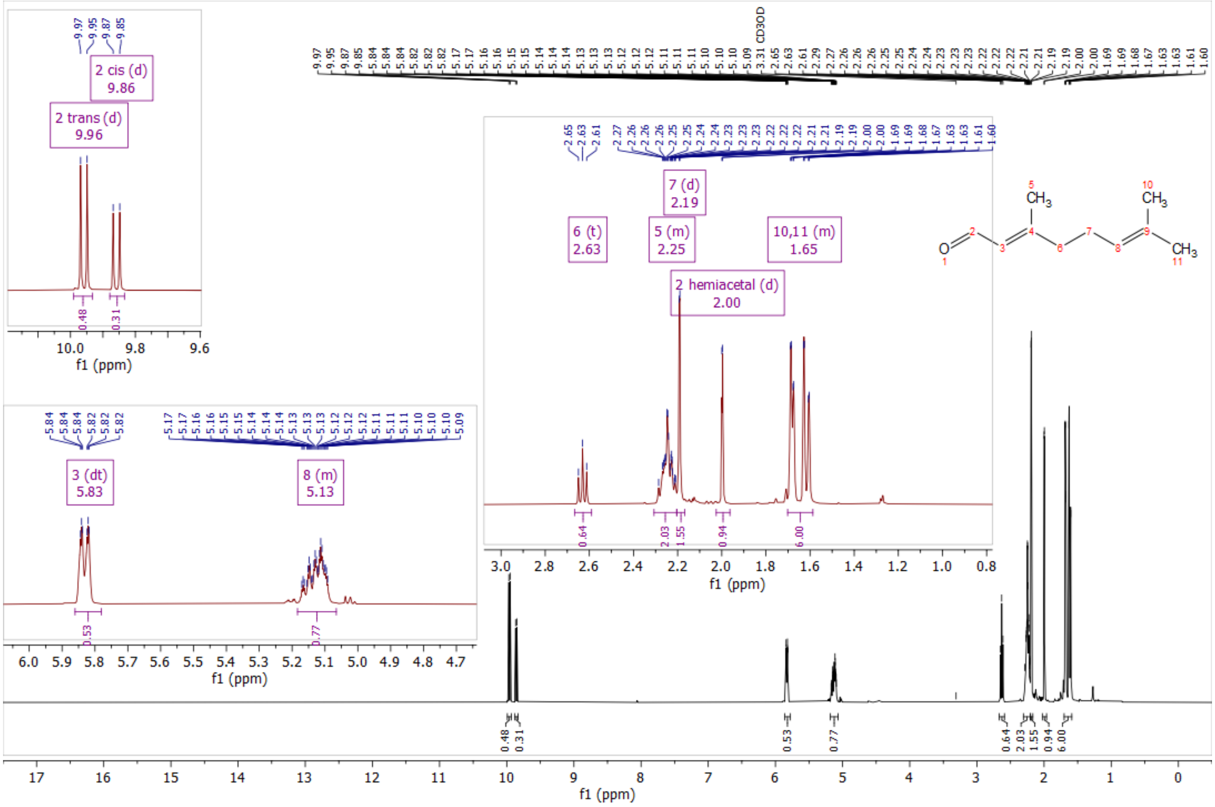

Figure S 12 ^{1}H NMR spectrum of citral in methanol-d4.

Signals of citral: δ H (400 MHz, methanol-d4) 1.59 – 1.70 (6 H, m), 2.00 (1 H, d, *J* 1.4), 2.19 (2 H, d, *J* 1.4), 2.20 – 2.31 (2 H, m), 2.63 (1 H, t, *J* 7.4), 5.07 – 5.18 (1 H, m), 5.83 (1 H, dt, *J* 8.1, 1.3), 9.86 (0 H, d, *J* 8.2), 9.96 (0 H, d, *J* 8.0).

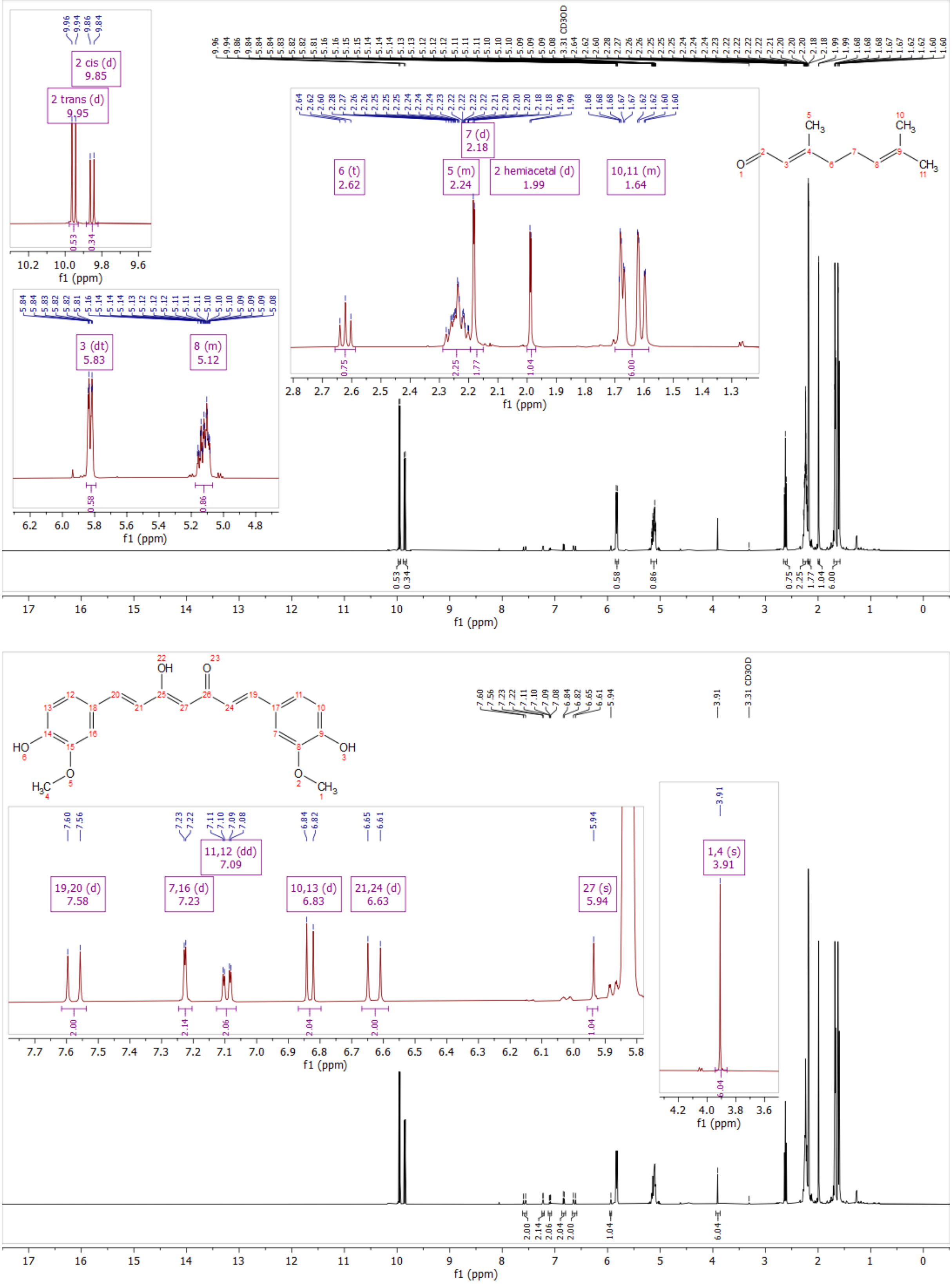

Figure S 13 ¹H NMR spectrum of curcumin in citral/methanol-d4 (30/70) (n/n) with assigned signals of citral (top) and of curcumin (bottom).

Signals of citral: δ H (400 MHz, methanol-d4) 1.58 – 1.70 (6 H, m), 1.99 (1 H, d, *J* 1.4), 2.18 (2 H, d, *J* 1.5), 2.19 – 2.29 (2 H, m), 2.62 (1 H, t, *J* 7.4), 5.07 – 5.17 (1 H, m), 5.83 (1 H, dt, *J* 8.0, 1.3), 9.85 (0 H, d, *J* 8.1), 9.95 (1 H, d, *J* 8.0).

Signals of curcumin: δ_{H} (400 MHz, methanol-d4) 3.91 (6 H, s), 5.94 (1 H, s), 6.63 (2 H, d, *J* 15.8), 6.83 (2 H, d, *J* 8.2), 7.09 (2 H, dd, *J* 8.3, 1.9), 7.23 (2 H, d, *J* 2.0), 7.58 (2 H, d, *J* 15.7).

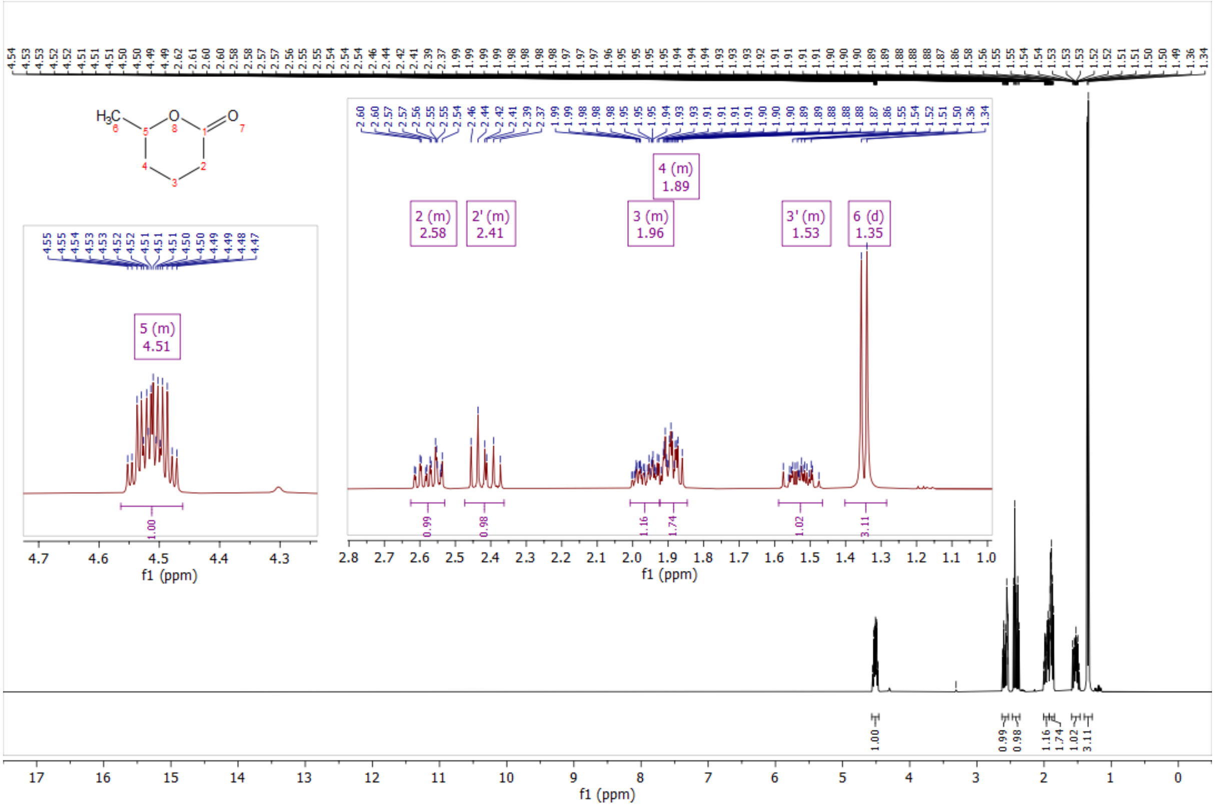

Figure S 14 ^{1}H NMR of delta-hexalactone in methanol-d4.

δ_{H} (400 MHz, methanol-d4) 1.35 (3 H, d, *J* 6.4), 1.47 – 1.59 (1 H, m), 1.85 – 1.92 (2 H, m), 1.92 – 2.01 (1 H, m), 2.36 – 2.47 (1 H, m), 2.53 – 2.63 (1 H, m), 4.46 – 4.56 (1 H, m).

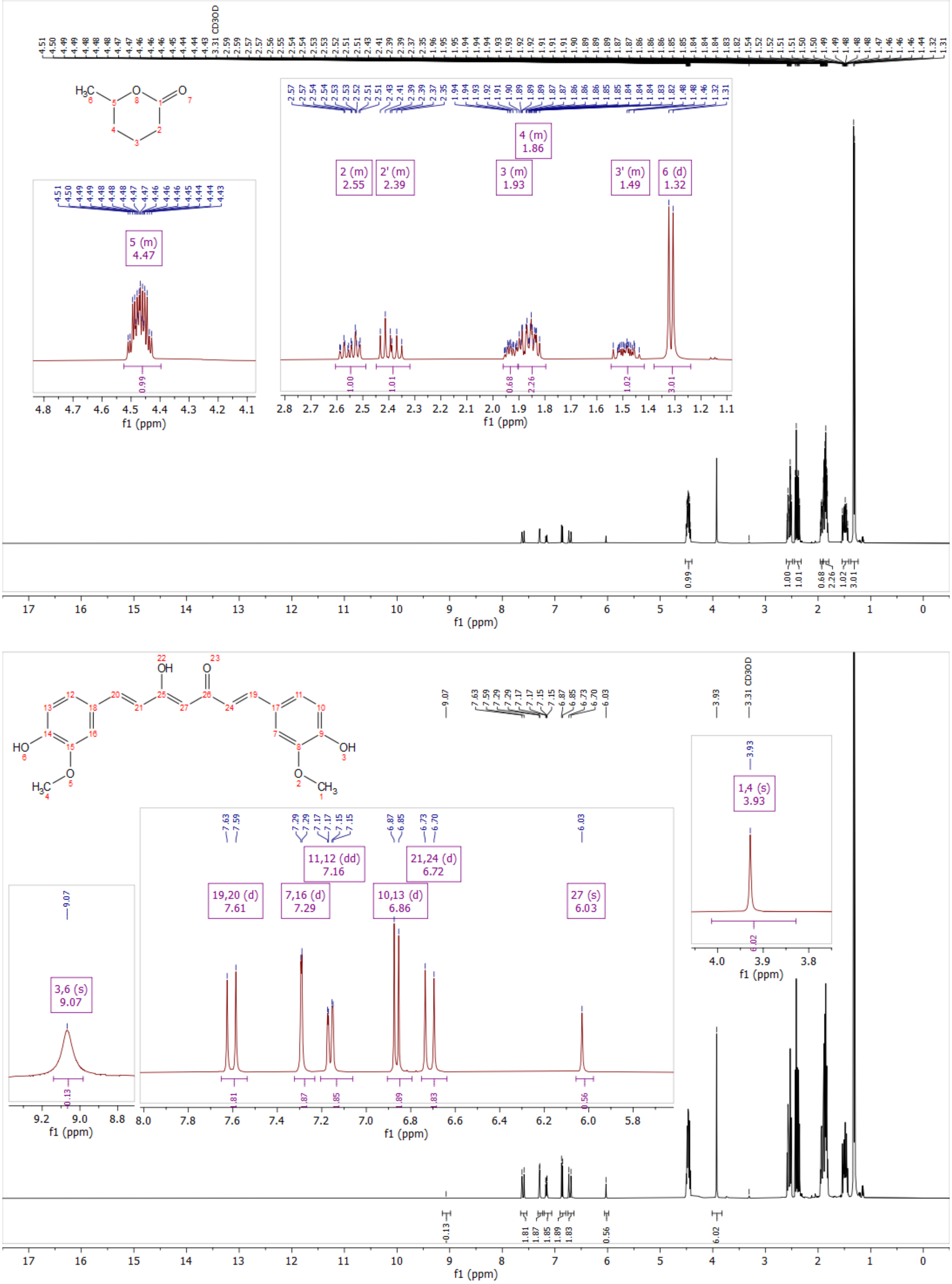

Figure S 15 ^{1}H spectrum of curcumin in delta-hexalactone/methanol-d4 (30/70) (n/n) with assigend signals of delta-hexalactone (top) and of curucmin (bottom).

δ H (400 MHz, methanol-d4) 1.32 (3 H, d, *J* 6.4), 1.42 – 1.55 (1 H, m), 1.80 – 1.90 (2 H, m), 1.90 – 1.96 (1 H, m), 2.32 – 2.45 (1 H, m), 2.49 – 2.61 (1 H, m), 4.40 – 4.52 (1 H, m).

δ H (400 MHz, methanol-d4) 3.93 (6 H, s), 6.03 (1 H, s), 6.72 (2 H, d, *J* 15.8), 6.86 (2 H, d, *J* 8.2), 7.16 (2 H, dd, *J* 8.3, 1.9), 7.29 (2 H, d, *J* 1.9), 7.61 (2 H, d, *J* 15.8), 9.07 (0 H, s).

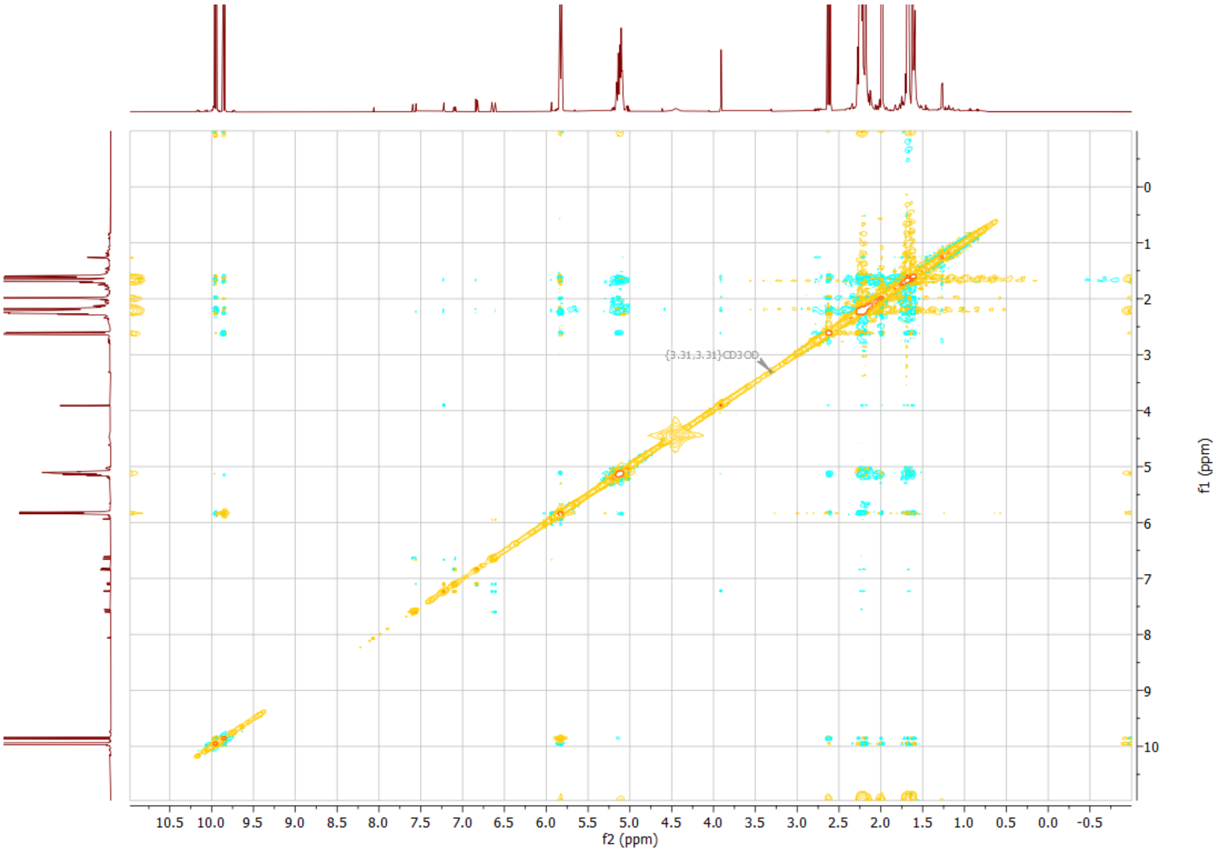

Figure S 16 ^{1}H-^{1}H NOESY spectrum of curcumin in citral/methanol-d4 (30/70) (n/n).

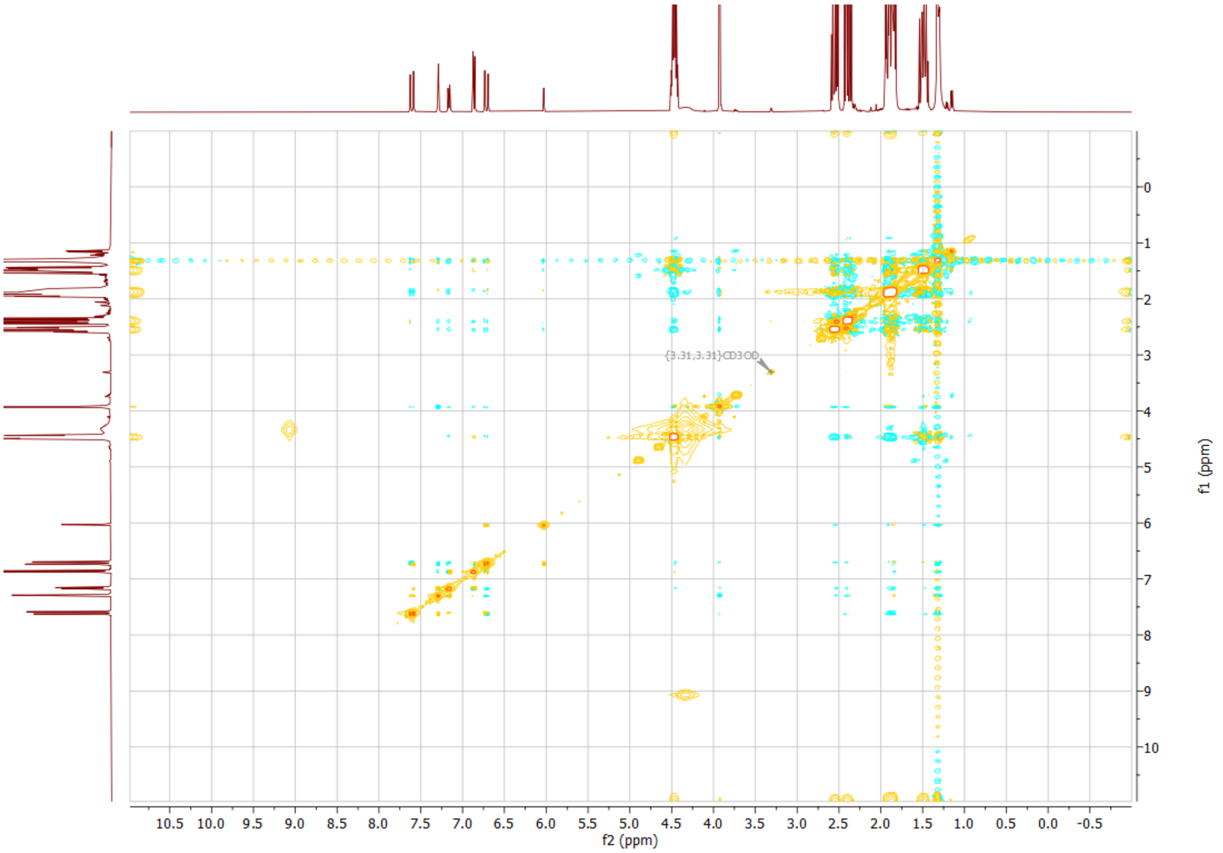

Figure S 17 ^{1}H-^{1}H NOESY spectrum of curcumin in delta-hexalactone/methanol-d4 (30/70) (n/n).
